# Supplementary material for: Association Analysis and Meta-Analysis of Multi-Allelic Variants for Large-Scale Sequence Data
Source: Genes (Basel). 2020 May 25;11(5):586. doi: 10.3390/genes11050586 (PMC7288273; doi:10.3390/genes11050586)
Supplement: Supplementary file 1 [file genes-11-00586-s001.zip › genes-805832-supplementary/multi-allelic-meta-analysis-Supplemental-Material-revision.docx]

**Supplementary Methods**

1. Bias in Naïve Analysis of Multi-allelic Variant.

We encode multi-allelic sites with $L$ alternative alleles as a $L$ vector $G=\left( G_{1},\ldots,G_{L} \right)$ with $G_{l}$ entry being the number of the $l^{th}$ alternative allele. It is tempting to analyze the association between the phenotype $Y$ and the number of $A_{l}$ alleles (i.e., $Y\sim G_{l}$). However, as we will point out below, this naïve analysis strategy compares the difference in the mean phenotype values between individuals that carry one copy of $A_{l}$ and the baseline group of individuals that carry no copy of $A_{l}$. For different $A_{l}$, the baseline group (i.e. individuals that carry no $A_{l}$ alleles) differs, which makes the effect estimates hard to interpret. It also makes it difficult to combine the estimates in gene-level association tests or in meta-analysis.

We first illustrate this with a simple numerical example. Consider a tri-allelic variant with two different alternative alleles $A_{1},A_{2}$, each with allele frequency of 0.2. Allele $A_{1}$ is causal. Carrying each additional copy of $A_{1}$ increases the mean value of $Y$ by 0.1 s.d.; Allele $A_{2}$ has no effect on the phenotype. If we perform a regression analysis between the phenotype and the number of $A_{2}$ alleles, i.e. $Y=\alpha+\beta_{2}G_{2}+\epsilon$, the parameter $\beta_{2}$ measures the change in the phenotype mean value per unit of change in the number of alternative alleles $A_{2}$, i.e. $\beta_{2}=E\left( Y | G_{2}=1 \right)-E\left( Y | G_{2}=0 \right)$. Below, we will show that $\beta_{2}\neq0$ even if the allele $A_{2}$ has no effect on the phenotype.

We note that $G_{2}=0$ include the genotype group of ${A_{0}}/{A_{0}},{A_{0}}/{A_{1}},{A_{1}}/{A_{1}}$, and $G_{2}=1$ include the genotype group of ${A_{0}}/{A_{2}},{A_{1}}/{A_{2}}$. Under the Hardy Weinberg equilibrium assumption, the genotype frequencies for $A_{0}/A_{0}$, $A_{0}/A_{1}$, $A_{1}/A_{1}$, $A_{1}/A_{2}$, $A_{0}/A_{2}$ and $A_{2}/A_{2}$ are equal to 0.36, 0.24 0.04 0.08 0.24 and 0.04. The conditional expectations $E\left( Y | G_{2}=1 \right)$ and $E\left( Y | G_{2}=0 \right)$ can be calculated as follows:

$$E\left( Y | G_{2}=1 \right)=\frac{E\left( Y | {A_{2}}/{A_{0}} \right)\Pr\left( {A_{2}}/{A_{0}} \right)+E\left( Y | {A_{2}}/{A_{1}} \right)\Pr\left( {A_{2}}/{A_{1}} \right)}{\Pr\left( {A_{2}}/{A_{0}} \right)+\Pr\left( {A_{2}}/{A_{1}} \right)}=0.025$$

$$E\left( Y | G_{2}=0 \right)=\frac{E\left( Y | {A_{1}}/{A_{0}} \right)\Pr\left( {A_{1}}/{A_{0}} \right)+E\left( Y | {A_{1}}/{A_{1}} \right)\Pr\left( {A_{1}}/{A_{1}} \right)+E\left( Y | {A_{0}}/{A_{0}} \right)\Pr\left( {A_{0}}/{A_{0}} \right)}{\Pr\left( {A_{1}}/{A_{0}} \right)+\Pr\left( {A_{1}}/{A_{1}} \right)+\Pr\left( {A_{0}}/{A_{0}} \right)}=0.05$$

So $\beta_{2}=-0.025$ even though the allele $A_{2}$ has no effect. Analyzing the association using naïve regression will lead to biased estimates of effects and inflated type I error (**Figure S1)**.

2. Description of the Participating Cohorts

SardiNIA study on aging (SardiNIA)

The SardiNIA study is a longitudinal, population-based study that includes 6,921 individuals, representing >60% of the adult population of 4 villages in the Lanusei valley on Sardinia (Italy)[1]. These individuals are clustered in 1,257 multigenerational families, up to 5 generations deep, and have been characterized for hundreds of quantitative traits. All participants gave informed consent to study protocols, which were approved by the Sardinian local research ethic committees: Comitato Etico di Azienda Sanitaria Locale 8, Lanusei (2009/0016600) and Comitato Etico di Azienda Sanitaria Locale 1, Sassari (2171/CE)) and by the NIH Office of Human Subjects Research as governed by Italian institutional review board approval. In association analysis, the covariates age, age^2, sex and status of former smoker were adjusted. A linear mixed model was used to perform the association analysis.

Metabolic Syndrome in Men Study (METSIM)

The METSIM study aims to investigate the metabolic syndrome, type 2 diabetes, cardiovascular disease, and cardiovascular risk factors[2]. It is an ongoing study of men aged 50 to 70 years, randomly selected from the population registry of the town of Kuopio, in Eastern Finland. In association analysis, the covariates age, age^2 and sex were adjusted. A linear mixed model was used to perform the association analysis.

Minnesota Center for Twin and Family Research (MCTFR).

The MCTFR sample is composed of two primary cohorts, a population-based sample of twins and their parents, and a sample of families with adopted children. The study design and genetic data have been described in detail in prior publications[2-5]. In association analysis, the covariates age, age^2 and sex were adjusted. A linear mixed model was used to perform the association analysis.

Center for Antisocial Drug Dependence (CADD)

The CADD is a study of over 8000 individuals from which participants were selected for genotyping[6]. Participants were selected for genotyping based on a measure of behavioral disinhibition taken in adolescence. Complete information on the genotyped sample and selection procedure have been described in detail previously[7]. In association analysis, the covariates age, age^2 and sex were adjusted. A linear mixed model was used to perform the association analysis.

Genes for Good

Genes for Good is an online study of the genetics of health and behavior (https://apps.facebook.com/genesforgood). Participation in Genes for Good is open to anyone over age 18 with a U.S. postal address. The covariates of age, age^2, sex, weight, height and 20 top principal components were adjusted. A linear mixed model was used to perform the association analysis.

COPDGene

COPDgene is a multisite observational study designed to research genetic factors that affect chronic obstructive pulmonary disease (COPD). Detailed information can be found in prior publications.[8] Individual European and African ancestries were analyzed separately. The covariates of age, age^2 and sex were adjusted. A linear mixed model was used to perform the association analysis.

**Table S1**. **The Power for Omnibus Association Tests with Multi-Allelic Sites.** We evaluated the power for single allelic and joint multi-allelic analysis as omnibus test for identifying associated variant sites. The power was compared under four different scenarios, 1) only the primary alternative allele is causal; 2) only one of the secondary alleles is causal; 3) all alternative alleles are causal with unidirectional effects and the genetic effects for causal alleles are simulated from $N^{+}\left( 0,\tau^{2} \right)$, and 4) all alternative alleles are causal with bi-directional effects and the genetic effects for causal alleles are simulated from $N\left( 0,\tau^{2} \right)$. The power for single-allelic and multi-allelic analysis as omnibus tests were evaluated by the fraction of replicates where the p-value of any alternative allele is < 5x10^-8^/1.1, a threshold that adjusts for the increased multiple testing burden due to multiple alternative alleles. The power for the collapsing approach was evaluated by the fraction of replicates where the p-value <5x10^-8^.

| Genetic Effects ($\boldsymbol{\tau}$) | Single-Allelic  Analysis | Joint Multi-Allelic  Analysis | Collapsing Multi-Allelic Sites |
| --- | --- | --- | --- |
| Only the Primary Alternative Alleles are Causal | | | |
| 0.1 | 0.49 | 0.5 | 0.44 |
| 0.25 | 0.75 | 0.76 | 0.71 |
| 0.5 | 0.86 | 0.87 | 0.83 |
| One of the Secondary Alternative Alleles are Causal | | | |
| 0.1 | 0.19 | 0.24 | 0.07 |
| 0.25 | 0.49 | 0.56 | 0.22 |
| 0.5 | 0.7 | 0.75 | 0.39 |
| All Alternative Alleles are Causal with Uni-Directional Effects | | | |
| 0.1 | 0.48 | 0.53 | 0.54 |
| 0.25 | 0.73 | 0.75 | 0.77 |
| 0.5 | 0.84 | 0.87 | 0.88 |
| All Alternative Alleles are Causal with Bi-Directional Effects | | | |
| 0.1 | 0.49 | 0.5 | 0.48 |
| 0.25 | 0.75 | 0.76 | 0.75 |
| 0.5 | 0.86 | 0.87 | 0.86 |

**Table S2**. **Top Signals from Gene-level Association Tests for All Genes**. We showed the top genes from the analyses using the burden test, SKAT and VT under two different minor allele frequency cutoffs (MAF<1% or MAF<5%). We also showed the number of rare variant sites under different minor allele frequency thresholds.

| **Gene** | **Statistic** | **P-value** | **Number of Variant Site with Rare Variants** | **Gene** | **Statistic** | **P-value** | **Number of Variant Site with Rare Variants** |
| --- | --- | --- | --- | --- | --- | --- | --- |
| **Burden Test with MAF<1%** | | | | **Burden Test with MAF<5%** | | | |
| MLKL | 16.81 | 4.1×10^-5^ | 28 | SHCBP1L | 25.41 | 4.6×10^-7^ | 27 |
| NPTX2 | 16.08 | 6.1×10^-5^ | 16 | LYZL6 | 16.72 | 4.3×10^-5^ | 6 |
| PM20D2 | 14.70 | 1.3×10^-4^ | 15 | EBF2 | 15.43 | 8.6×10^-5^ | 18 |
| **SKAT Test with MAF<1%** | | | | **SKAT Test with MAF<5%** | | | |
| PNP | 904166 | 2.2×10^-5^ | 8 | PNP | 904166 | 2.2×10^-5^ | 8 |
| ABTB1 | 1654137 | 5.5×10^-5^ | 28 | CSDA | 2502957 | 9.6×10^-5^ | 28 |
| CSDA | 2502957 | 9.6×10^-5^ | 28 | ABTB1 | 1834396 | 0.000149 | 29 |
| **VT Test with MAF<1%** | | | | **VT Test with MAF<5%** | | | |
| TTC15 | 21.98 | 1.9×10^-5^ | 27 | SHCBP1L | 25.41 | 3.6×10^-6^ | 27 |
| SCMH1 | 21.40 | 3.6×10^-5^ | 17 | TTC15 | 21.98 | 2.5×10^-5^ | 27 |
| TSKU | 20.53 | 5.9×10^-5^ | 15 | SCMH1 | 21.40 | 2.6×10^-5^ | 17 |

**Table S3: Single Variant Association Results for Variants within Identified Gene-level Associations in Table 5 and Table S2. (See excel spread sheet in another file).**

**Figure S1. Bias and Type I Error of Naïve Regression Analysis of** $\boldsymbol{Y}$ **over the Genotype of Allele** $\boldsymbol{A}_{\boldsymbol{2}}$**.** We consider a multi-allelic variant with two different alternative alleles $A_{1},A_{2}$. The reference allele $A_{0}$ has frequency 0.5. The allele $A_{1}$ is causal. Carrying each additional copy of $A_{1}$ increases the mean value of $Y$ by 0.1 s.d. The $A_{2}$ allele has no effect on the phenotype. In panel (A), we plot the bias of the genetic effect estimates against the frequency allele $A_{2}$. In panel (B), we plot the type I error for the regression analysis against the allele frequency of $A_{2}$ using the significance level $\alpha=0.05$. Depending on the allele frequency, the bias can be as large as 0.05 and the type I error can be as large as 0.2.

**
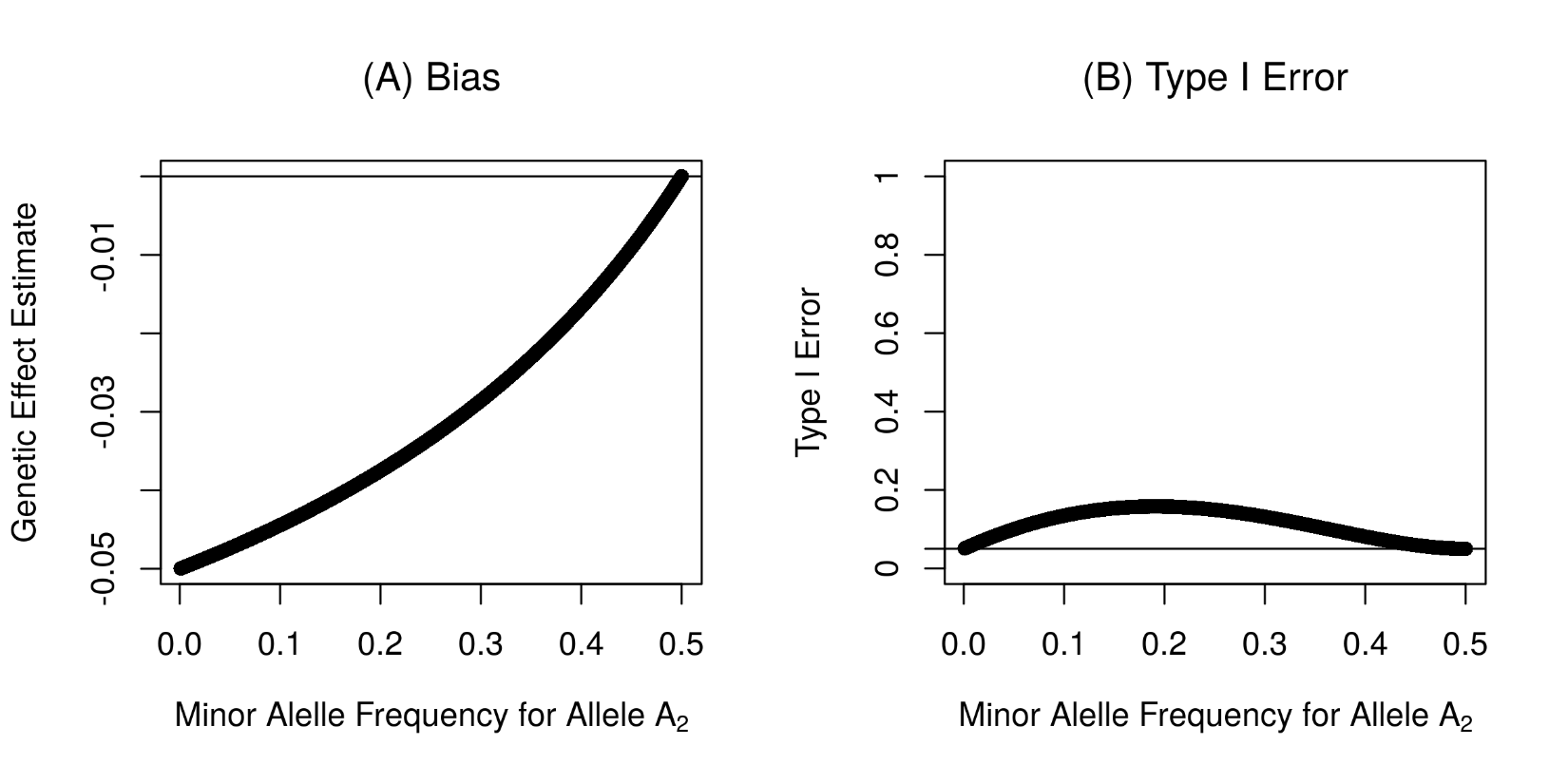
**

**Figure S2: Hardy-Weinberg Equilibrium P-value Distribution for Genome-wide SNPs (Panel A) and Multi-allelic Variants (Panel B).** Almost all SNPs have insignificant HWE p-values who supported the validity of the association results.


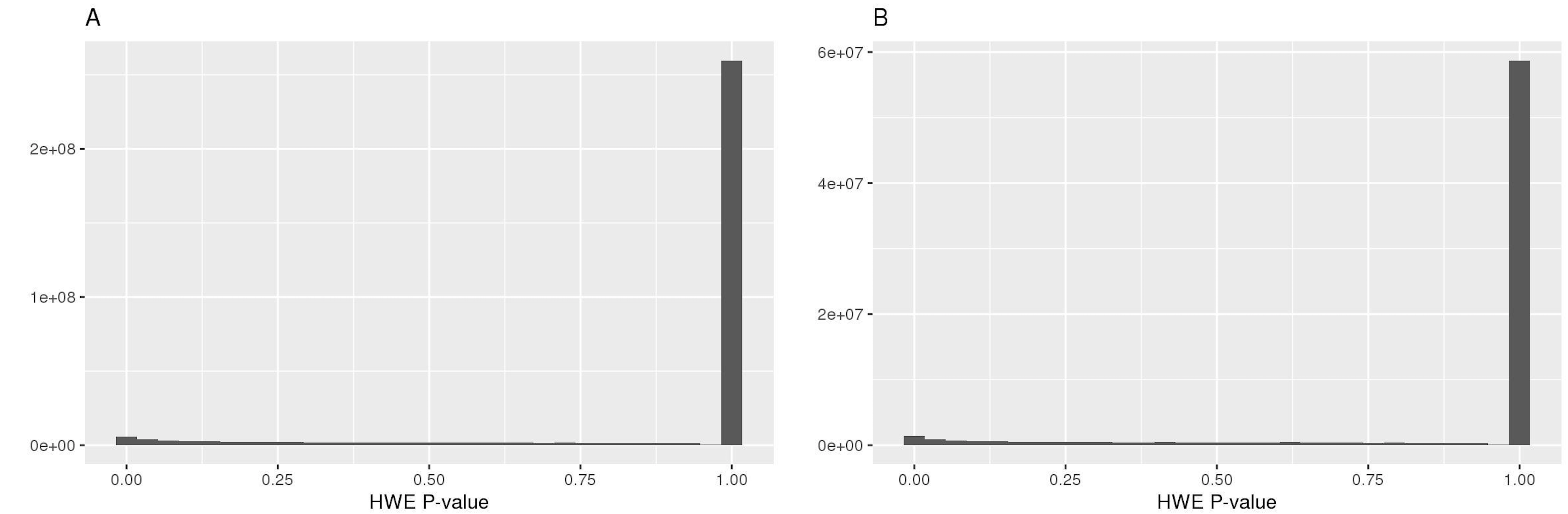


**Figure S3: Histograms of Allele Frequencies from Genome-wide Variants (Panel A) and Multi-allelic Variants (Panel B).**

**
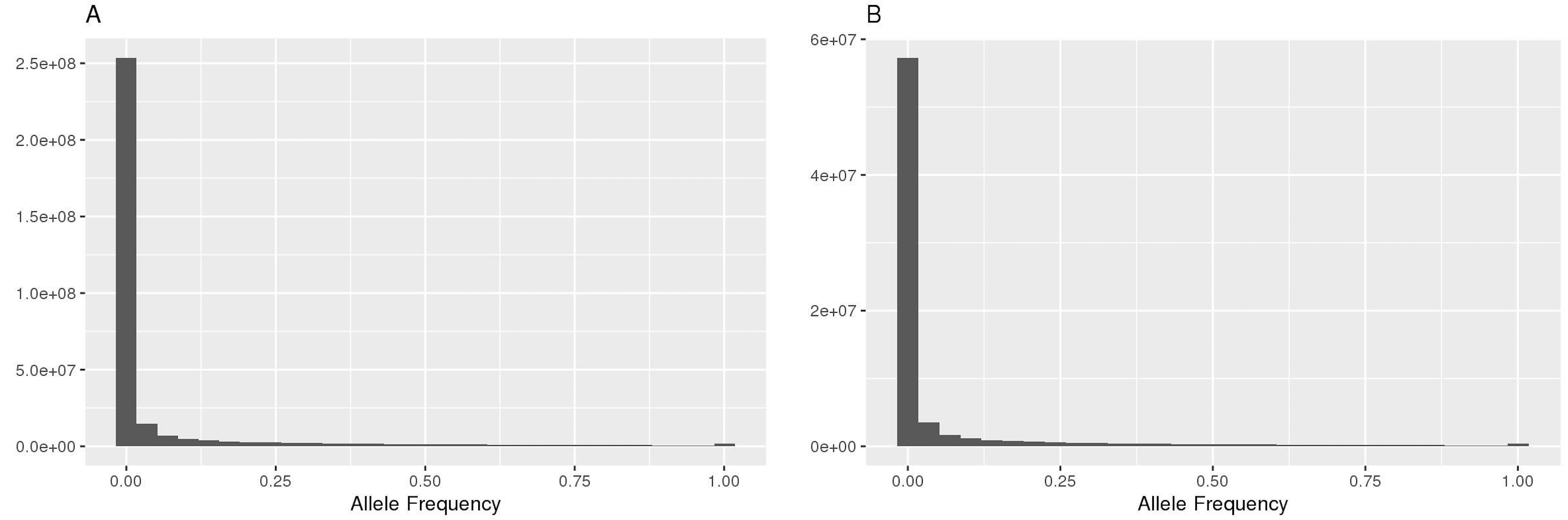
**

**Figure S4** **Quantile-Quantile Plot for Meta-Analysis Results for Single Variant Association Test of Multi-Allelic Variants**. Panel (A) displays the results for joint multi-allelic analysis, and panel (B) displays the results for single-allelic analysis. The QQ plot is stratified by allele frequency bins $\left( 0,0.001 \right], (0,001,0.01]$ and $(0.01,1]$. The genomic control inflation factor $\lambda$ is calculated separately for each frequency bin.


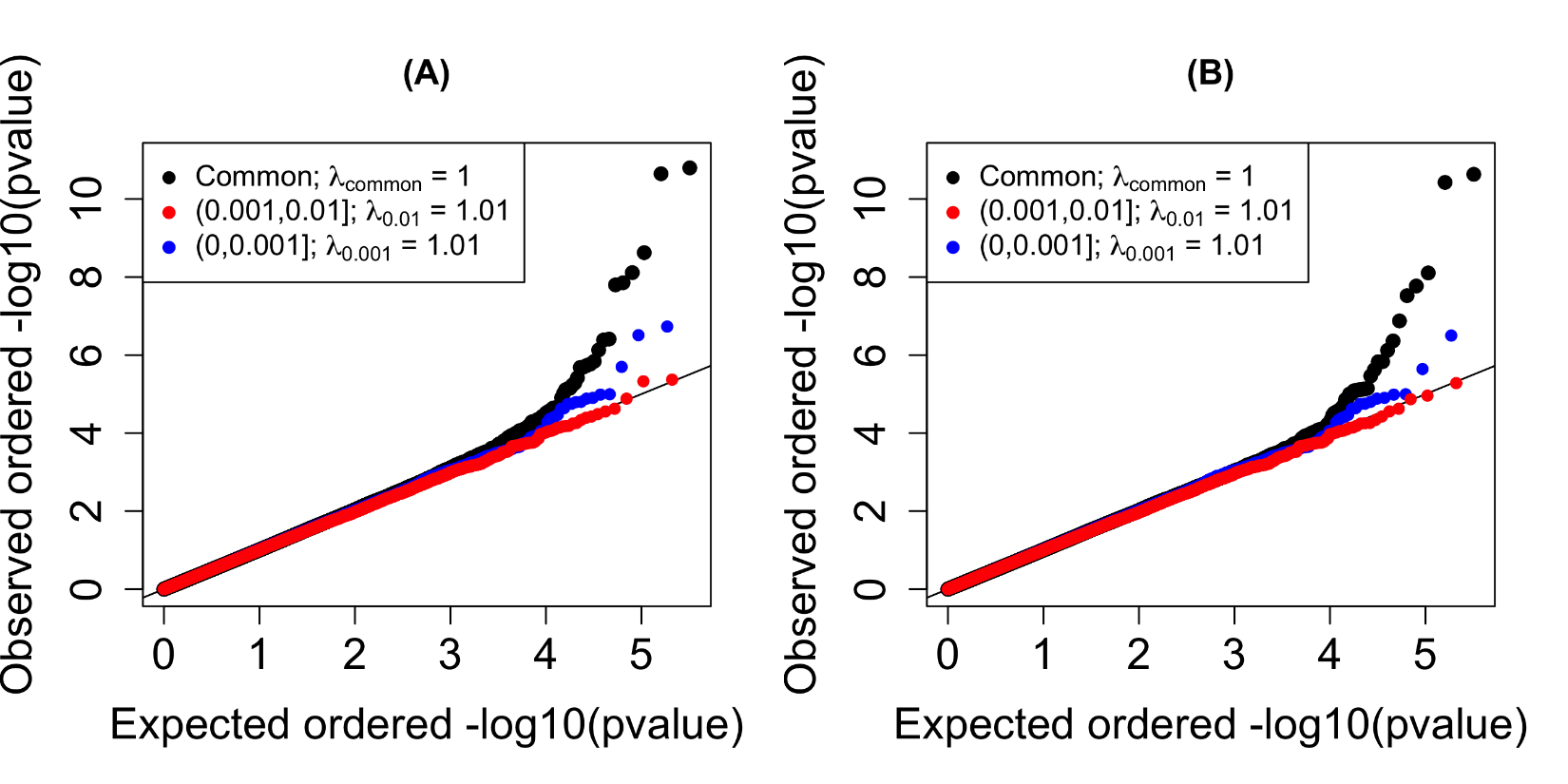


**Figure S5**: **Manhattan Plot for Meta-Analysis Results for Single Variant Association Test for Multi-Allelic Variants.** Panel (A) displays the results for joint multi-allelic analysis, and panel (B) displays the results for single-allelic analysis.


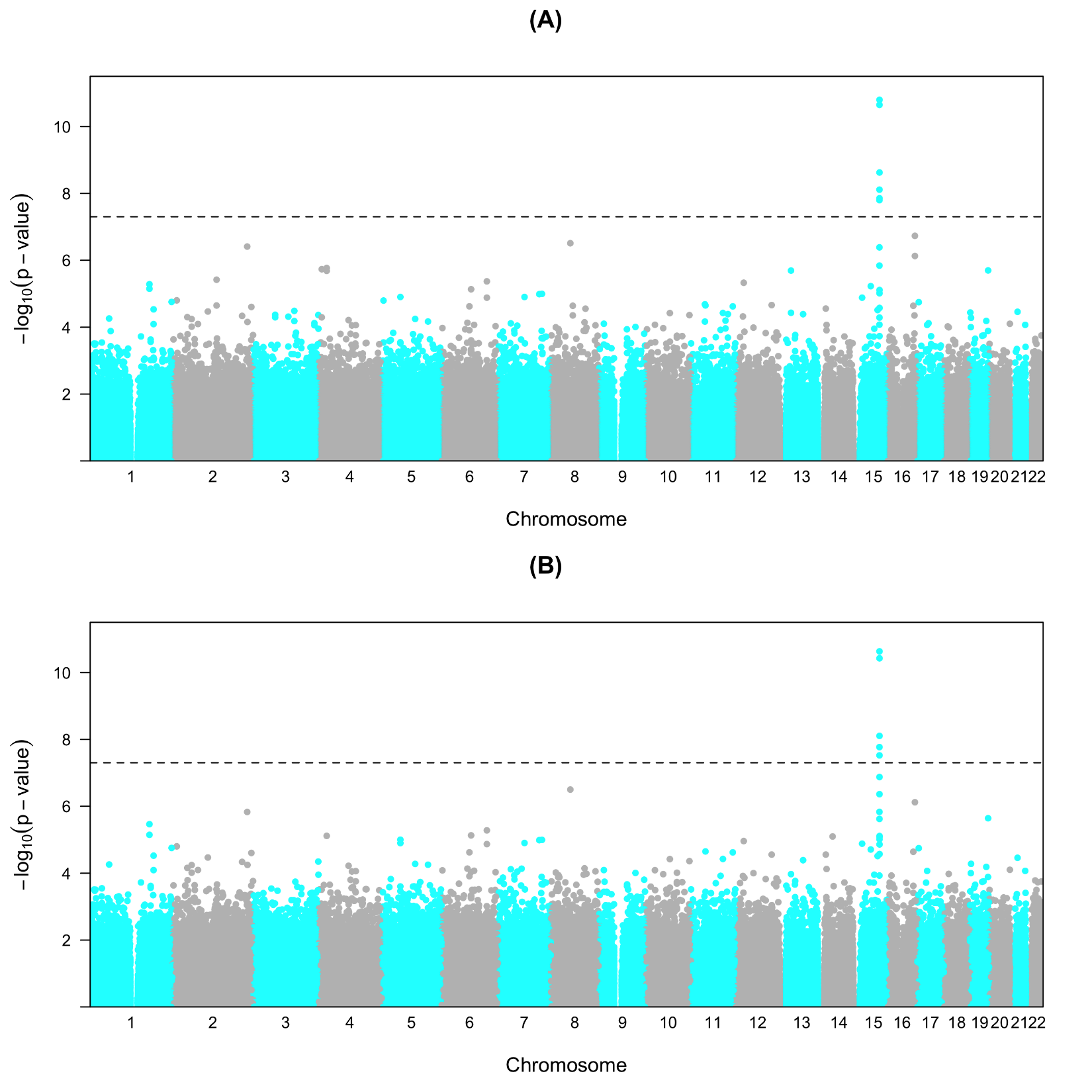


**Figure S6**. **Comparison between Single-allelic and Joint Multi-allelic Analysis in the Meta-analysis of Cigarettes-Per-Day Phenotype.** The comparison is stratified by common (MAF>1%) and rare variants (MAF<1%). The $-\log_{10} (p-value)$ is plotted for all multi-allelic variants. Each dot represents the p-value for the effect of one allele in single allelic (x-axis) and joint multi-allelic analysis (y-axis).


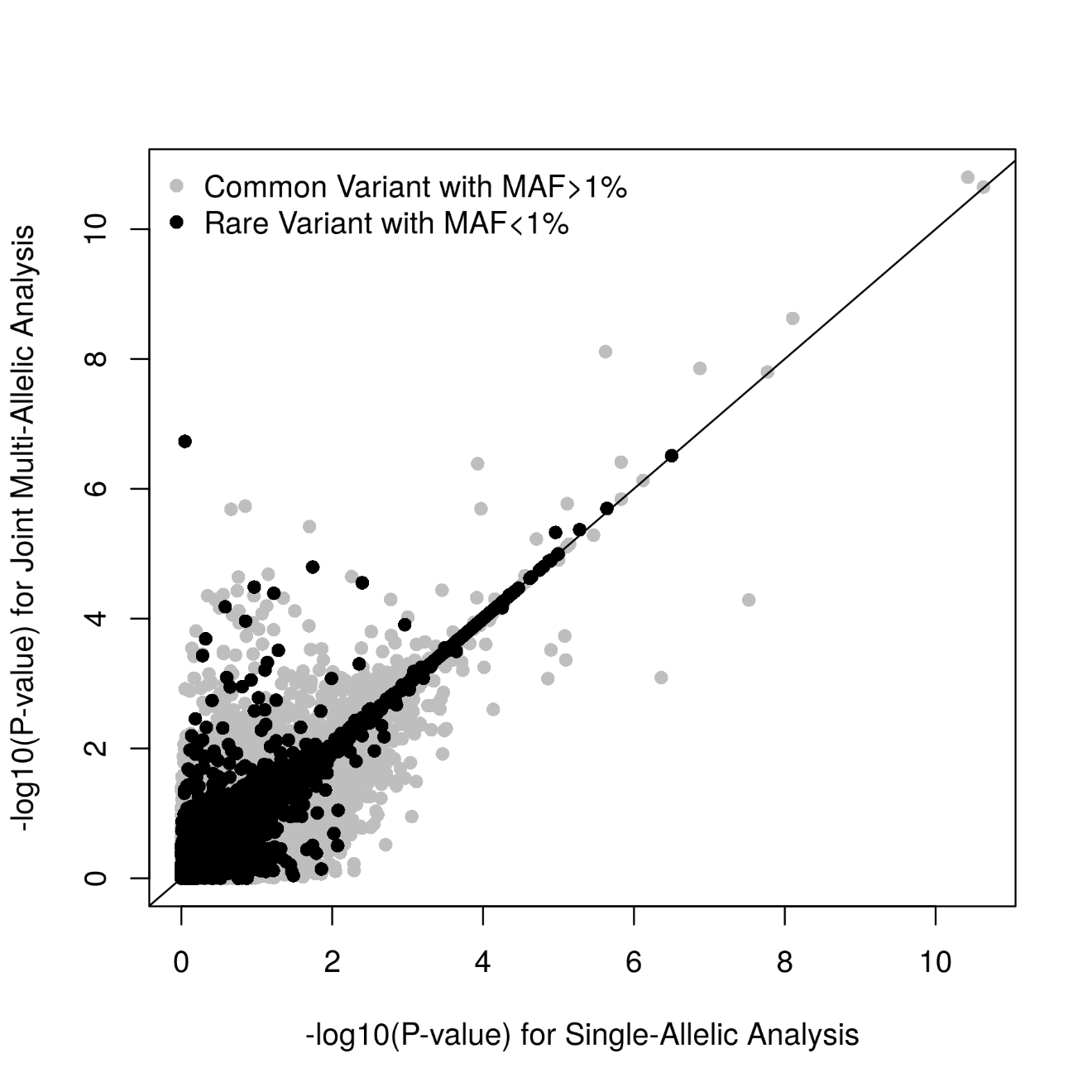


**Figure S7**. **Quantile-Quantile Plot for Gene-level Association Test for Genes with Rare Alleles at Multi-Allelic Variant Sites**. We show the QQ plot for genes with rare alleles at multi-allelic variant sites. Three rare variant association tests (simple burden, SKAT and VT) were conducted under two different minor allele frequency thresholds (MAF<0.01 and MAF<0.05). The genomic control inflation factor for all tests was well controlled (<1.05).


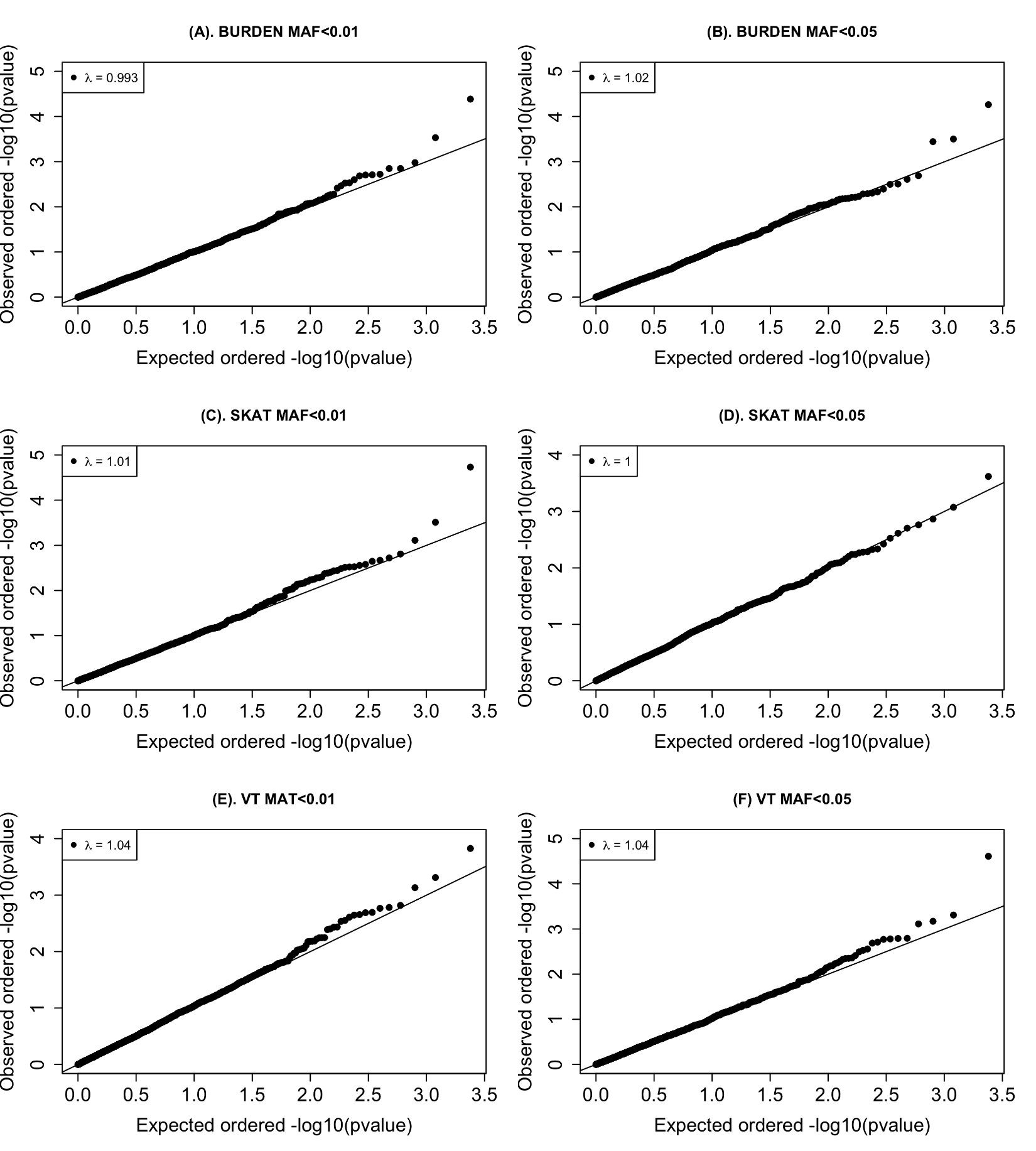


**Figure S8**. **Quantile-Quantile Plot for Gene-level Association Test for All Genes.** Three rare variant association tests (simple burden, SKAT and VT) were conducted under two different minor allele frequency thresholds (MAF<0.01 and MAF<0.05). The genomic control inflation factor for all tests was well controlled (<1.05).


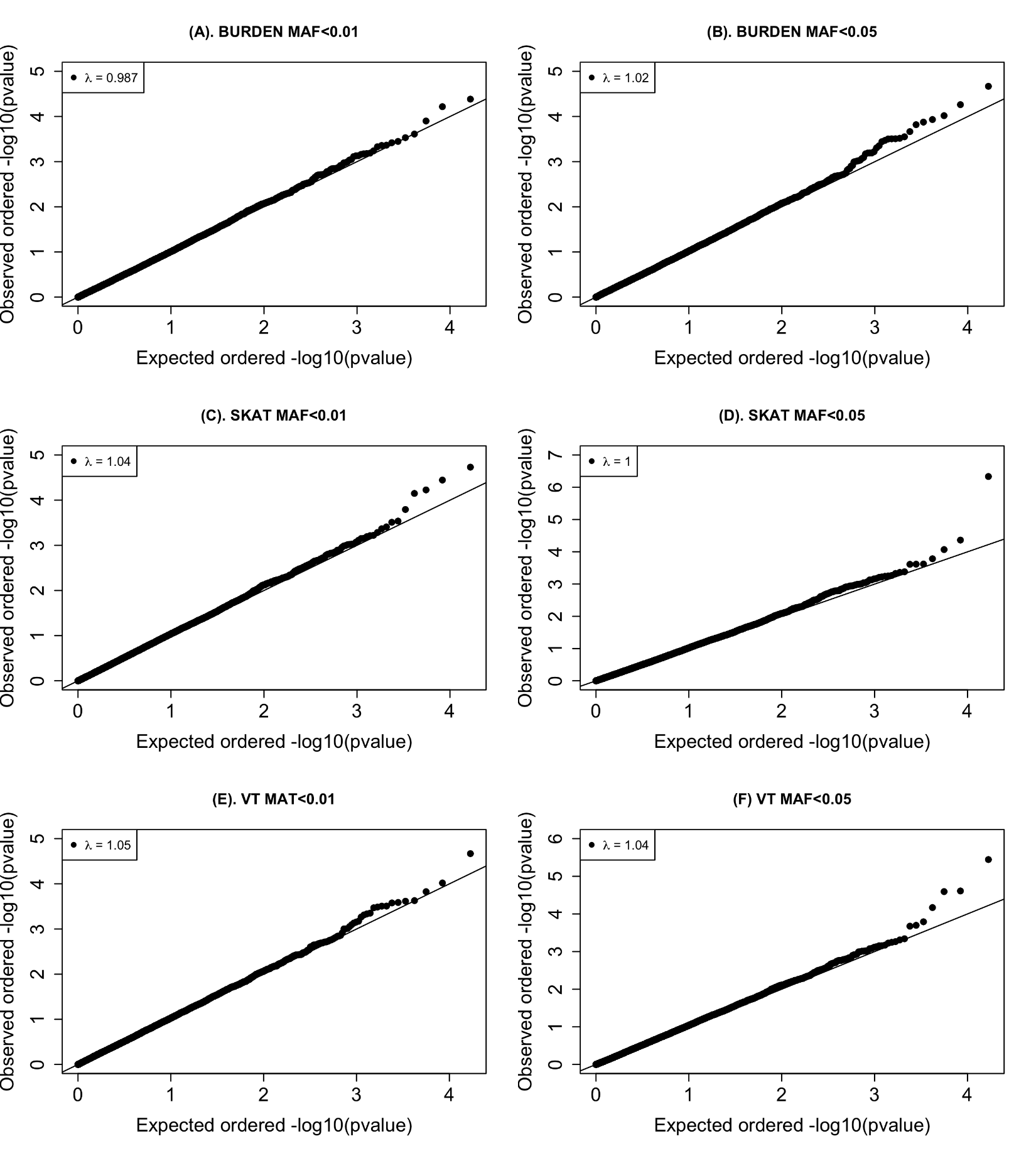


**Figure S9. Comparison of Results for the Joint Multi-allelic Analysis and the Analysis that Discards Multi-allelic Sites.** We plotted the –log_10_(p-values) for (A-B) BURDEN test with MAF<1%, 5% (C-D) SKAT test with MAF<1%,5% and (E-F) VT test with MAF<1%, 5%.


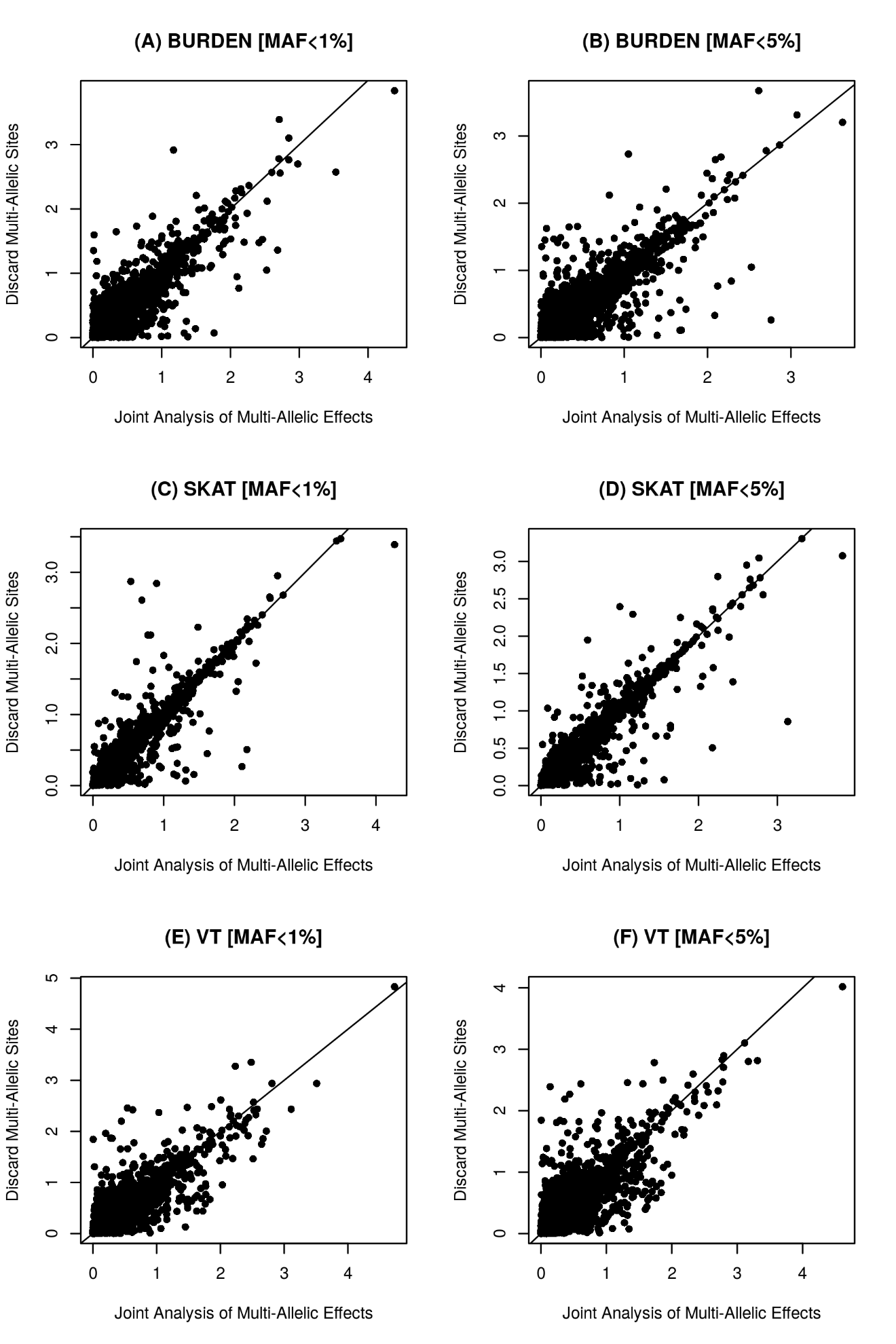


**REFERENCE**

1. Pilia, G.; Chen, W.M.; Scuteri, A.; Orru, M.; Albai, G.; Dei, M.; Lai, S.; Usala, G.; Lai, M.; Loi, P., et al. Heritability of cardiovascular and personality traits in 6,148 Sardinians. *PLoS genetics* **2006**, *2*, e132, doi:10.1371/journal.pgen.0020132.

2. Stancakova, A.; Javorsky, M.; Kuulasmaa, T.; Haffner, S.M.; Kuusisto, J.; Laakso, M. Changes in insulin sensitivity and insulin release in relation to glycemia and glucose tolerance in 6,414 Finnish men. *Diabetes* **2009**, *58*, 1212-1221, doi:10.2337/db08-1607.

3. Miller, M.B.; Basu, S.; Cunningham, J.; Eskin, E.; Malone, S.M.; Oetting, W.S.; Schork, N.J.; Sul, J.H.; Iacono, W.G.; McGue, M. The Minnesota Center for Twin and Family Research genome-wide association study. *Twin Research and Human Genetics* **2012**, *15*, 767-774.

4. Iacono, W.G.; McGue, M.; Krueger, R.F. Minnesota Center for Twin and Family Research. *Twin Research and Human Genetics* **2006**, *9*, 978-984, doi:10.1375/183242706779462642.

5. Iacono, W.G.; McGue, M. Minnesota Twin Family Study. *Twin Res* **2002**, *5*, 482-487.

6. Stallings, M.C.; Corley, R.P.; Dennehey, B.; Hewitt, J.K.; Krauter, K.S.; Lessem, J.M.; Mikulich-Gilbertson, S.K.; Rhee, S.H.; Smolen, A.; Young, S.E., et al. A genome-wide search for quantitative trait Loci that influence antisocial drug dependence in adolescence. *Arch Gen Psychiatry* **2005**, *62*, 1042-1051, doi:10.1001/archpsyc.62.9.1042.

7. Derringer, J.; Corley, R.P.; Haberstick, B.C.; Young, S.E.; Demmitt, B.A.; Howrigan, D.P.; Kirkpatrick, R.M.; Iacono, W.G.; McGue, M.; Keller, M.C., et al. Genome-Wide Association Study of Behavioral Disinhibition in a Selected Adolescent Sample. *Behav Genet* **2015**, *45*, 375-381, doi:10.1007/s10519-015-9705-y.

8. Regan, E.A.; Hokanson, J.E.; Murphy, J.R.; Make, B.; Lynch, D.A.; Beaty, T.H.; Curran-Everett, D.; Silverman, E.K.; Crapo, J.D. Genetic epidemiology of COPD (COPDGene) study design. *COPD* **2010**, *7*, 32-43, doi:10.3109/15412550903499522.
